# What’s cooking? The normalization of meat in YouTube recipe videos consumed by South Asian British Muslims

**DOI:** 10.1080/15528014.2023.2196195

**Published:** 2023-04-11

**Authors:** Gilly Mroz, Hibba Mazhary, James Painter

**Affiliations:** a Department of Zoology, University of Oxford, Oxford, UK; b School of Geography, University of Oxford, Oxford, UK; c Reuters Institute for the Study of Journalism, University of Oxford, Oxford, UK

**Keywords:** Meat, diet, Youtube, television, cookery, British muslims

## Abstract

Muslim consumers in the UK eat more meat than the national average. Individuals from ethnic minority backgrounds, particularly South Asian communities, experience poorer health outcomes, including diabetes and cardiovascular disease, associated with meat consumption. According to a YouGov survey, British Pakistani and Bangladeshi consumers use television cookery programs and social media (particularly YouTube) as their main digital sources of dietary information. Against this background, this study uses a mixed-method approach to show how meat is normalized in YouTube recipe content. Using quantitative analysis of 77 recent recipe videos presented by four leading British chefs (Jamie Oliver, Gordon Ramsay, Nigella Lawson and Nadiya Hussain) and halal recipe videos, we find that meat-based recipes overwhelmingly outnumber vegetarian/vegan ones, and that, whereas environmental or animal welfare concerns are hardly mentioned, health narratives feature in some videos. Using critical discourse analysis of a sample of videos, we show how meat consumption is rationalized by the “absenting” of meat’s animal origins (making it “normal”), the “defaultization” of meat (making it “natural” and “necessary”), and “positive emotional routines” (making it “nice” and “necessary”). We consider how these representations of meat serve to overcome the “meat paradox” and legitimize, and thereby normalize, meat consumption among British Muslims.

## Introduction

Despite growing interest in plant-based diets in western countries (Giraud 2021), meat remains a normal and accepted part of society. According to a 2021 YouGov survey conducted for this study, 9% of adults in the UK identify as vegan or vegetarian; by extension, it can be assumed that 91% of individuals continue to eat meat to some extent. Meat consumption is particularly high among the British Muslim community, with individuals eating more meat per capita than the national average (Stannard and Clarke 2020). This is supported by the YouGov survey, which found that fewer British South Asians identify as vegan or vegetarian (8% of British Pakistanis and 5% of British Bangladeshis) than those of white ethnicity (9%).

### Cultural context

Discussions around Muslim meat-eating are informed by various religious, cultural, socio-economic and political factors that contribute to its normalization. Meat is associated with Islamic ritual practice, with animal sacrifice being a devotional exercise, particularly during the yearly festival of *Eid-ul-Adha* (Friedlander 2020; Tayob 2019). According to Oleschuk, Johnston, and Baumann (2019), meat elimination is particularly challenging for some Muslims because of the risk of “being perceived as disrespecting or rejecting their culture or religion” (p. 350). Whilst recognizing the importance of animal sacrifice in Islamic ritual practice, there is also evidence of Muslim consumers mobilizing religious arguments to moderate meat consumption (Robinson 2014; Istasse 2015) and to emphasize the religious imperative for environmental sustainability and animal welfare (Pettinato 2016; MacGregor, Walker, and Katz-Gerro 2019). Socially, meat is an important part of special occasions and hosting for British Muslims (Stannard and Clarke 2020; Isakjee and Carroll 2021). Oleschuk, Johnston, and Baumann (2019) describe how meat acts as a “cultural vessel containing social connections, rituals and traditions” for ethnic minority communities (p. 348). A political context also shapes Muslim meat consumption in the UK, with discussions of halal meat and slaughter often intertwined with a politics of belonging and questions of national identity (Isakjee and Carroll 2021; Lever 2020). Early Muslim settlers in the UK, most arriving post-WW2 from the Indian subcontinent (Lewis 1994), had informal networks of halal meat procurement, sourcing and slaughtering the animals themselves (Lever 2018). The halal meat industry became increasingly consolidated from the 1980s onwards (Lever 2018); it is now fast-growing, with halal meat widely available (Stannard and Clarke 2020).

### Health inequalities

The lower level of meat reduction or elimination among British Muslims is also influenced by the context of significant social and financial inequality (Muslim Council of Britain 2021). Research has shown that lower-income groups suffer worse health outcomes than those with higher incomes (Choi et al. 2020). Individuals from Pakistani and Bangladeshi communities have long experienced some of the poorest health outcomes in the UK (Raleigh and Holmes 2021). For example, Bangladeshi men are almost four times more likely, and Pakistani men three times more likely, to be diagnosed with type 2 diabetes, compared with the general British male population (Sproston and Mindell 2006). Diabetes is a risk factor for cardiovascular disease; accordingly, the incidence of myocardial infarctions (heart attacks) is higher in South Asians than in non-South Asians (Scarborough et al. 2010).

Studies have found meat consumption to be associated with increased risk of diabetes (Tonstad et al. 2013) and heart disease (Tong et al. 2019), and red and processed meat consumption associated with increased risk of mortality with regard to cardiovascular disease (Wang et al. 2016). However, it is important to note that differences in genetics (such as body shape and fat storage) and lifestyle factors (such as higher consumption of fried or oily foods using ghee, high salt consumption, smoking and/or chewing tobacco, and lack of exercise) can affect the incidence of diabetes and CVD among different ethnic groups (Scarborough et al. 2010; Sproston and Mindell 2006; Gupta 2022).

### The “meat paradox”

While health is regularly given as the main reason an omnivore would consider reducing or eliminating meat from their diet, environmental factors and animal welfare concerns play an important supplementary role (Mullee et al. 2017; Jones 2021). Despite these arguments, meat consumption levels remain high in western society. This is reflective of the “meat paradox,” where consumers enjoy eating meat while disapproving of the suffering or killing of animals (Loughnan, Haslam, and Bastian 2010; Bastian and Loughnan 2016). For those unwilling to turn to vegetarianism or veganism to overcome this cognitive dissonance, researchers point to a variety of moral disengagement “strategies” which serve to justify meat-eating (Graça, Manuela Calheiros, and Oliveira 2014).

The main direct (conscious) strategy is rationalization, which involves “providing reasonable justifications for one’s behavior” (Piazza et al. 2015) (p. 114). This closely aligns with Joy (2010)’s concept of the “3 Ns” in justifying meat consumption: the idea that meat is “natural” (a part of human history and biology), “normal” (an accepted social norm), and “necessary” (an integral part of a healthy human diet). Piazza et al. (2015) have added a fourth N, “nice,” to this rationalization framework, since meat-eaters often refer to the taste of meat, and/or to the pleasure they derive from it, in justifying their consumption. Linked to the idea of meat as “natural” is the strategy of speciesism – the belief that animals are inferior to humans (Rothgerber 2013)—and the denial of animal suffering, including the denial of animals’ cognitive abilities in order to downplay this suffering (Brock et al. 2011).

The main indirect (unconscious) strategy is avoiding thinking about animal suffering (Rothgerber 2013). It is supported by disassociation – that is, suppressing the knowledge of meat’s animal origins (Kunst and Hohle 2016). Other scholars have used different terms to describe this strategy. Joy (2010) discusses the “invisibility” of systems of animal exploitation, while Mills (2017) comments that “the animal experience that precedes this end [cooked] product is overwhelmingly invisible” (p. 186). Arcari (2017), drawing inspiration from Adams (2010)’s notion of the “absent referent,” uses the term “absenting,” with animals being “de-animated, objectified and aggregated” (p. 74). Mills (2017) also draws on the idea of “de-animation,” suggesting that language (and, we add, images) can “aggregate, materialize and otherwise treat [animals] as an inanimate resource” (p. 82), for example in the use of the term “beef” instead of “cow.” Accordingly, products where the animal has been de-animated, and flesh has been “de-animalized,” such as hamburgers, are a popular food choice (Kubberød et al. 2002).

### Cookery content and consumers

The 2021 YouGov survey found that social media and television cookery programs were the two main media types that influenced the eating habits of British South Asian Muslims. Social media was the main source among individuals with Pakistani heritage (27%), followed by television cookery programs (19%); for individuals with Bangladeshi heritage this was slightly lower (at 10% and 11%, respectively). Respondents who answered that social media influenced their eating habits were then asked to specify which platforms: YouTube was the most popular among individuals of Bangladeshi heritage (63%) and second most popular among those of Pakistani heritage (40%).

The role of YouTube as a source of information about food for British Muslims is supported by other statistics. As part of the Stannard and Clarke (2020) study, 765 British Muslim consumers were asked: “On a scale of 1 to 5 how appealing are the following potential future ideas within the halal meat industry;” 60% found “YouTube video recipes” appealing.^1^ Moreover, a report by Ofcom (2020) found that YouTube was the third most popular website or app used by minority ethnic groups in the UK (26%), for any reason, after only the BBC (56%) and Google (43%).

The importance of television cookery programs and YouTube for shaping consumer dietary choices is well established. According to Barnes (2017), celebrity chefs are a form of “talking label” in that “they act as both a cultural intermediary and boundary object to construct knowledge around choosing/shopping, cooking and eating and connect audiences to food and themselves” (p. 170). Their influence on the public’s eating habits has grown with the advent of social media. Goodman and Jaworska (2020) observed that Jamie Oliver, Gordon Ramsay and Nigella Lawson were the most influential of their 33 identified “digital food influencers” (DFIs), thanks to their having the largest number of Twitter followers (>6 million for Oliver, 5 m for Ramsay, and 2 m for Lawson). Their presence on YouTube is equally extensive: as of November 2021, Oliver had 5.6 m subscribers, Ramsay had 18.4 m, and Lawson had 295k.^2^ Oliver himself recently shared his preference for YouTube as a source of cookery information over television (Rawlinson 2021). Given the appeal of cookery content among British Muslims, whose interest in British cuisine is second only to South Asian cuisine (Stannard and Clarke 2020), and given the popularity of these three chefs (in addition to Nadiya Hussain, the UK’s most prominent Muslim celebrity chef) among the general UK population, we can assume a substantial reach by these culinary celebrities among members of the British Muslim community.

### Research questions

Against this background, this paper aims to explore how recipe content available on YouTube and widely consumed (though not necessarily created) by British Muslims – with a focus on British Pakistani and Bangladeshi communities – contributes to the normalization of meat consumption. Although we focus on the portrayal of meat as “normal,” we also consider the portrayal of Piazza et al. (2015)’s other 3 Ns in support of this: the idea that meat is “natural,” “necessary” and “nice.” We aim to answer three main research questions:

1. What is the dietary distribution of videos? Do more videos contain meat or vegetarian/vegan recipes?

2. To what extent are narratives pertaining to the impact of meat or plant-based eating on health, the environment, or animal welfare presented?

3. What strategies or techniques are used in the videos to normalize meat for viewers?

## Materials and methods

This study relied on a mixed method approach and was undertaken in three parts: data collection, quantitative analysis and critical discourse analysis. We focus on media consumed by British Muslims due to their high meat consumption rates and social, financial and health inequalities. We refine this further to Muslims of Pakistani and Bangladeshi heritage because together they comprise the largest ethnic group of British Muslims (52.9%) (Muslim Council of Britain 2015), using data about these groups available from the 2021 YouGov survey.

Survey results showed that social media (particularly YouTube) and television cookery programs influence the way British Pakistani and Bangladeshi Muslims describe their eating habits. We examined British recipe content available on YouTube due to the platform’s popularity among these two ethnic groups, the wide variety of cooking content available on it (much of which is also present on UK television), and the interest in British cuisine by Muslims in the UK, second only to South Asian cuisine (Stannard and Clarke 2020). We chose videos which focus on demonstrating recipes, rather than those containing more general food- and/or cooking-related content, to refine the scope of this work and to facilitate consistency in our analysis.

The website YouTube Data Tools^3^ was used to retrieve lists of the most popular and/or relevant YouTube videos during a five-year period between January 2016 and December 2020 according to two research strands: British celebrity chefs and British halal content.

1. British chefs. Four search terms were used: “Jamie Oliver,” “Gordon Ramsay,” “Nigella Lawson” and “Nadiya Hussain.” These chefs were chosen due to their popularity among, and influence on, the British public (Goodman and Jaworska 2020), and prominence on UK television, with all having hosted and continuing to present a wide array of cookery programs. Oliver and Lawson first appeared on British television in 1999 in *The Naked Chef* and *Nigella Bites*, respectively, while Ramsay made his first television appearance in 2004 in *Ramsay’s Kitchen Nightmares*; Hussain is a more recent television personality, having won *The Great British Bake Off* in 2015, and is a popular British Muslim chef of Bangladeshi heritage. Many clips from these and other television cookery programs are available on YouTube, whether published by the channels of British broadcasters (such as BBC and All4) or on the chefs’ personal channels (Hussain does not have her own YouTube channel, but is present in numerous videos posted particularly by the BBC). The lists of videos for these search terms were retrieved by view count in order to focus our analysis on the most widely consumed recipe videos by these chefs.

2. British halal search. The search term “halal recipes” was used. This was chosen due to the higher proportion of cooking- and recipe-related content generated by this search query in comparison to others, such as “halal food,” “halal cooking” and “Muslim recipes.” This search also helped to capture the more amateur food influencer content popular on YouTube to complement the professional chefs’ videos. The country code “UK” was included in order to retrieve videos more relevant to and/or popular among the British Pakistani and Bangladeshi Muslim public (“UK” and “GB” returned similar results). This was an important qualifier, since the majority of videos retrieved by using “halal recipes” without this country code were more relevant to East and South East Asian Muslim communities, and therefore less pertinent to this study. These videos were retrieved according to relevance, as the more niche nature of the search term meant that this method yielded more videos than by view count. Once retrieved this way, the videos were sorted by view count.

Having downloaded the lists of YouTube videos (on 5 August 2021 for the chefs, 17 August 2021 for the halal content), the researchers agreed on inclusion and exclusion criteria to ensure only relevant videos were retained for analysis. The focus had to be a recipe for a main dish or meal intended for use by the audience, rather than other aspects of cookery programs. We excluded sauces, dips and side dishes, but retained desserts and baked goods at this stage, primarily due to the density of these recipes among Hussain’s most viewed videos. As such, videos which only briefly featured a dish being cooked (e.g., as part of a travel video or program), or which did not feature a recipe at all (e.g., when food was discussed or eaten, rather than cooked), were excluded. For the television chefs, dishes had to be cooked by each respective chef, though this could be with a guest chef. Videos whose titles indicated recipes containing pork (whether explicit, such as “pork belly,” or more implicit, such as “carbonara” or “spam”) were also omitted, based on the unlikelihood of British Muslims choosing to watch these due to pork’s religious prohibition and cultural taboos (Armanios and Ergene 2018); videos whose titles did not specify the type of meat (such as “meatballs” or “lasagna,” or compilation videos) were retained. The 20 videos with the highest view counts that fit these criteria were gathered from the data retrieved for each of the five search terms. This number was chosen as it provided us with a robust sample for our quantitative analysis and a selection of the most popular videos on which to base our critical discourse analysis. Once these were gathered, videos containing recipes for desserts and baked goods were removed to facilitate, and simplify, our analysis around the normalization of meat. Our final dataset consisted of 77 recipe videos (Oliver, *n* = 16; Ramsay, *n* = 19; Lawson, *n* = 14; Hussain, *n* = 9; halal recipes, *n* = 19).

Two researchers undertook quantitative analysis to identify the types of dishes being cooked. Each video was categorized into one of three groups: “meat,” “vegetarian/vegan (v/vg)” and “mixed (meat and v/vg).” “meat” included both land-based meat and seafood. The “mixed” category was largely used for compilation-style videos that contained clips of both meat and v/vg recipes. Observations from previous research about the nature of the recipe video genre (Mills 2017), as well as the focus and duration of these videos, suggested that they would not feature many health, environment and/or animal welfare narratives. However, we decided to assess the presence or absence of these narratives, as they are key reasons an omnivore might reduce or eliminate meat from their diet, or a vegetarian/vegan would continue following a meat-free diet (Jones 2021; Mullee et al. 2017).

Critical discourse analysis (CDA) was then undertaken. Analysis focused on three techniques that contribute to the normalization of meat in the recipe videos: the absenting of animal origins, the “defaultization” of meat, and positive emotional routines. These were identified deductively, based on previous studies exploring cognitive dissonance and moral disengagement strategies concerning meat eating (Graça, Manuela Calheiros, and Oliveira 2014; Rothgerber 2013; Bastian and Loughnan 2016; Piazza et al. 2015; Arcari 2017), and inductively, based on recurring images and tropes observed during the earlier steps. A small selection of videos was chosen for the CDA to facilitate in-depth analysis. As CDA is a subjective form of analysis, our selection was purposive for the analysis of our chosen meat normalization techniques, rather than representative of our entire dataset. Each video had to feature a recipe for land-based meat. Where possible videos containing one standalone recipe were chosen; however, recipes as part of compilation videos were admitted where we found them particularly illustrative of our chosen themes. At least one halal recipe video is discussed for each technique, and at least one video from each chef is discussed across our analysis.

## Results

### Quantitative analysis

#### Video distribution

Table 1 shows the distribution of our 77 videos containing savory recipes based on their content. 73% contained meat recipes (including both land-reared meat and seafood) while 14% contained vegetarian or vegan recipes. 13% contained mixed (meat and v/vg) content.

**Table 1.** Distribution of videos by dominant food type.

	Jamie Oliver (*n*=16)		Gordon Ramsay (*n*=19)		Nigella Lawson (*n*=14)		Nadiya Hussain (*n*=9)		Halal recipes (*n*=19)		Total (*n*=77)	
Meat	10	63%	17	89%	7	50%	5	56%	17	89%	56	73%
Vegetarian/vegan (v/vg)	4	25%	0	0%	3	21%	4	44%	0	0%	11	14%
Mixed - meat and v/vg	2	13%	2	11%	4	29%	0	0%	2	11%	10	13%

Ramsay had the highest proportion of meat-only recipe videos, at 89%, and was the only chef to have *no* vegetarian/vegan (v/vg) videos. 11% of his videos contained both meat and v/vg recipes. The other chefs also had more meat-based videos than v/vg ones, but the ratios were far closer. Oliver had 63% of his videos as meat-only and 25% as v/vg, with the rest mixed, while Lawson had 50% meat-only and 29% v/vg, with the rest mixed. Hussain had the closest ratio, with 56% meat-only and 44% v/vg, and no mixed-content videos.

The data for the halal recipes mirrored that of Ramsay: 89% of the videos were meat-only, 11% were mixed, and none were v/vg. This may be due to the search term, “halal recipes,” with “halal” tending to be used in reference to meat.

#### Narratives

No references were made to the environment, while the only references to animal welfare were in Oliver’s qualification of his use of “free-range” eggs. Health was mentioned in 11 videos, dominated by Ramsay (4) and Oliver (3). Meat described as healthy was limited mainly to chicken. This was not without contestation. For example, Ramsay explains to his children that “chicken is healthy, but not fried, every night.”^4^ Lawson comments that “scientific tests of chicken soup” have shown it to possess “anti-inflammatory and antibacterial properties,” though she does not cite her sources.^5^ For Hussain, the “healthiness” of chicken is mentioned only in a video title, “Healthy Chicken Shawarma.”^6^ For halal content creator Immy Maryam, health is associated with weight loss, as she describes her chicken and vegetable dish as “meal prep to lose weight and get shredded.”^7^ Health narratives around turkey and fish only appeared once each, but without contestation: Ramsay’s butcher describes turkey as “a great lean meat,”^8^ while Hussain describes her salmon poké bowl as “nutritious.”^9^

Although Ramsay also discusses the benefits of plant-based foods – such as oats, nuts and berries in his Bircher muesli recipe^10^—Oliver’s positive health narratives concern only vegetarian and vegan food. He says of his “Veggie Spaghetti Bolognese,” for example: “it’s really nutritious, it’s perfectly balanced, it’s three of your five fruit and veg a day, which is fantastic. And it’s bigging up wonderful ways to get your protein without eating meat.”^11^

Full details of how all the health narratives appeared in the videos can be found in the Supplementary Material.

### Critical discourse analysis

We identify three main (indirect) strategies in our recipe videos which contribute to the normalization of meat. The first is the absenting of meat’s animal origins. Very few television cookery programs discuss animal welfare or depict industrial-scale meat production (Mills 2017). According to Hopkins and Dacey (2008), people enjoy these programs for “the creativity, the sensuousness, the clever techniques,” but would be “horrified” to see a lamb brought in and killed (p. 579). As we have seen, our data supports these observations: there are no references to animal welfare, nor any representations of the meat industry. Moreover, we find that the chefs and cooks rarely mention, and even more seldom discuss, the animal origins of the meat they are cooking, for example which part of an animal a cut of meat came from.^12^ While these various forms of absenting are widespread, our focus here is on the visual presentation of meat as another medium which distances meat from its animal origins and, therefore, legitimizes its consumption.

The second is the “defaultization” of meat in dishes, where meat is unexpected or unspecified. Consumers often regard meat consumption as natural according to human history and biology: we evolved to eat it and it is something our bodies crave (Piazza et al. 2015). As a result, meat is often considered a “default” option for meals; indeed, many dishes, such as burgers and lasagnes, have become synonymous with meat, and in such cases qualifiers are usually required to indicate a plant-based, rather than default meat, version. This tendency also persists thanks to the perception that meat-based options follow “the path of least resistance” (Joy 2010) (p. 106); in contrast, plant-based options are often perceived as more difficult, or the “resistant choice” (Happer and Wellesley 2019) (p. 131).

The third is evoking what have been described as positive “emotional routines” (Arcari 2017), with a focus on social occasions, domestic bliss and indulgence. This is particularly true of recipes featuring in television cookery programs, which invite audiences to “take pleasure in meat” (Mills 2017) (p. 190). By associating meat with these three forms of emotional routine, these programs remind the viewer just how “nice” it is to eat meat and thus rationalize its consumption.

## Absenting of animal origins

### The appearance of meat

In many cases meat first appears already cut into pieces or minced, and it is often difficult, even impossible, to distinguish which part of the animal (or, indeed, which animal) the meat originated from. In Hussain’s “Easy Chicken Tikka Masala,”^13^ we first see the chicken meat, boneless and cut into chunks on a ceramic plate; she then spoons them into her frying pan, in which she is cooking onions, garlic and spices. Though still recognizable as chicken meat at this stage, with its fleshy, light pink, almost translucent appearance, there is no reference to which part of the chicken it is from. An adjacent pan contains the same ingredients, but further along in the cooking process. Now the animal origins are even less recognizable. The chicken pieces are cooked through, the rawness removed; they are opaque and coated in thick spices. The distancing of chicken-as-meat from chicken-as-animal continues as Hussain adds tomato soup to the dish, engulfing the meat. Thanks to these three transformative steps – cooking the meat, coating it in spices and smothering it in sauce – its animal origins have become unrecognizable.

Another example is found in “How to cook Half Moon Pies Recipe” by halal blogger Cook with Anisa.^14^ As with Hussain, we first see the raw chicken meat in a dish; however, rather than a handful of large pieces of chicken meat resting on a homely, ceramic dish, the chicken is more finely cut and appears crammed into a Pyrex bowl, as though awaiting a laboratory experiment. Hussain’s three transformative steps are also taken further in this recipe. When added to the frying pan, the raw chicken meat becomes white and opaque. But, having been chopped so finely, it appears granular and mince-like, and the chicken pieces blend in with the other ingredients of similar size – diced onions and peppers, and sweetcorn kernels. They coat one another, transforming into a mass of indistinct ingredients. Anisa then smothers the mixture with a béchamel sauce, rendering all the contents unrecognizable. But there is one further step: encasing the mixture in pastry. We are thus viewing not a chicken dish, but a pastry dish (indeed, there is no mention of chicken in the video title).

While chicken is used as a vessel for flavor in Hussain’s recipe, its status in the half moon pies recipe is reduced even further to just another ingredient. This has the effect of non-differentiation and, by extension, normalization. The techniques implemented in the videos – cooking, coating, smothering and encasing – transform the animal flesh into just another “inanimate resource” (Mills 2017) (p. 82). This not only absents the animal origins of the chicken meat, but even (particularly in Anisa’s recipe) obscures the meat content of the dish. The further along the transformative process the meat is taken, the easier it is for the consumer to disassociate meat from its animal origins and legitimize its consumption.

## “Defaultization” of meat

### Unexpected meat

Although most video titles accurately describe the contents of the recipe, a number are misleading, particularly when meat features in a recipe that the viewer might assume to be vegetarian. An example is Oliver’s “Ultimate Mac & Cheese”^15^ which, unexpectedly, contains pork – an everyday ingredient for Oliver, but impermissible for the Muslim viewer. Following the placing of the mac and cheese dish in the oven, the viewer might expect no further steps to be required. But the video now zooms in on a chopping board on which Oliver is slicing a small stack of bacon rashers, before focusing on a frying pan in which the chopped bacon is sizzling alongside herbs and garlic. He adds breadcrumbs, and the mixture, continuing to fry gently, turns golden. When he serves a portion of the mac and cheese onto a plate, he sprinkles the bacon mixture on top. Although the macaroni pasta and cheese sauce are the crux of the dish, we now only see them protruding out from under a blanket of bacon *pangritata*.

According to Joy (2010), “[p]ractically and socially, it is vastly easier to eat meat than not meat” (p. 106). But Oliver is already making a vegetarian recipe; the additional step of cooking bacon – typically considered not an essential component of a mac and cheese dish – subverts the concept of meat as easy and the meat-free option as difficult. This additional task suggests that meat is not only normal, but a natural, and even necessary, component of any savory dish.

### Unspecified meat

In some videos there is no indication of what type of meat being cooked. In “Halal English Breakfast” by Zeinah Nur,^16^ she does not mention what meat the sausages or bacon in her dish are made from. When she cooks her bacon – traditionally made from pork – she says, anticipating the viewer’s surprise, “yes, bacon; but it’s halal bacon.” The qualifier “halal” implies that her bacon and sausages are made from meat (most likely turkey or chicken). Despite vegetarian alternatives being readily available in British supermarkets, her decision to replace pork with other halal meat suggests that meat remains the default option for the dish.

When she places the bacon onto the frying pan, we hear it start instantly to sizzle; we then see the bacon up close, sizzling, but still relatively raw, before seeing it darker and crispier in the next clip. A similar process occurs with the sausages. At the end when we see the final dish, the unappetizing, raw meats have been transformed into glistening bacon and golden sausages, plated alongside hash browns, tomato, omelet, toast and baked beans. Although the plating suggests that bacon and sausages are of equal importance with the other ingredients, the video as a whole implies that meat – regardless of what animal it comes from – is a natural and necessary component of an English breakfast. This modification of a quintessentially British national dish to make it halal-compliant echoes Armanios and Ergene (2018)’s description of “halal cuisine,” where diaspora Muslims adapt certain recipes to “integrate into various national and global landscapes” (p. 212). The “Halal English Breakfast” recipe is therefore an assertion of a distinctly Muslim British identity, tempered by the fact that the type of meat in the bacon and sausage remains unspecified, making the difference less noticeable and retaining the familiarity of the dish.

## Positive emotional routines

### Social occasions

Meat-eating is strongly linked with “key rituals of sociality” (Arcari 2017) (p. 81), where meat-based meals represent “acts of love or friendship” (Happer and Wellesley 2019) (p. 130). This is especially palpable in Hussain’s “insane BBQ lamb ribs” recipe.^17^ In the opening scenes, family and friends are seen at a social gathering happily devouring her lamb ribs. The guests are of different ethnicities (South Asian and white) and ages (adults and children), which recommends this dish as a celebratory feast that unites people of all backgrounds. Lamb has a particularly important role in social occasions for British Muslims: they have a much higher lamb consumption than the national average year-round and there is a sharp increase in demand for lamb around religious festivals (Stannard and Clarke 2020).

As with her chicken tikka masala recipe, discussed above, the lamb ribs undergo a transformation: Hussain jokes that, not unlike partygoers, they “are going to get so dressed up for this party you won’t even recognize them.” Indeed, they first appear beige and unappetizing, before becoming crispy and tender and, most importantly, covered in a sticky glaze. Whereas in her curry recipe the chicken is engulfed by sauce, here the lamb is enhanced by it, glistening on a large serving tray. This lamb dish has echoes of the “showstopper” centerpieces Hussain baked on her way to winning *The Great British Bake Off*, an association which evokes the idea of meat as just as integral to a social gathering as a cake.

The presence of meat at social occasions also features in “Ramadan Dishes for Suhoor and Iftar” by Tasty.^18^ One of the presenters, Bim, discusses a typical Nigerian “potato and egg” dish eaten for Suhoor (the pre-dawn meal during Ramadan) which she says can contain sardines, corned beef or sausages. Whereas it is the visual, “showstopper” aspect of Hussain’s lamb that evokes positive emotions, in this video the narrative content fulfills this purpose. As Bim explains:

*I love Ramadan every year. It makes me feel really connected, like there are literally millions of us across the world all doing the same thing at the same time and it’s really heart-warming and it makes me incredibly happy, like I’m really part of a global community.*

Bim’s commentary supports Oleschuk, Johnston, and Baumann (2019)’s description of meat as a “cultural vessel containing social connections, rituals and traditions, and bonds between individuals, families, and communities” (p. 348). This cultural repertoire acts as a “social lubricant” to enable consumers to navigate the meat paradox (p. 338), not only by being enjoyable, but also by playing an important role in cultural preservation among ethnic minority communities.

### Domestic bliss

According to Stannard and Clarke (2020), 81% of British Muslims consider mealtimes important family occasions. The idea of family connection over food is closely associated with the comfort of the domestic space. This is perhaps clearest in the recipe videos of Lawson, the self-dubbed “domestic goddess,” for example in her “Homemade Spaghetti and Meatballs.”^19^ The concept of domestic bliss, in this case in the form of maternal care, is instantly apparent: in the first scene, while entering the kitchen and tying an apron around her waist, Lawson explains that “pasta and meatballs are what I make when I want to shimmer into Italian mamma mode.” Having added grated parmesan, an egg, semolina and seasoning to a Pyrex bowl of minced pork and beef, she mixes it together with her hands before forming teaspoon-sized meatballs; their diminutive size and dainty appearance suggests they are being made with her children in mind. This notion is supported by Lawson’s warm smile as she forms the meatballs and places them on a tray, as though contemplating the joy of cooking for her children.

The idea of domestic bliss is heightened when we see Lawson and her two children, in the living/dining space, rolling pasta dough through the pasta maker as a fire crackles in the fireplace behind them. Once the pasta is made, Lawson returns to the kitchen; she places the meatballs into the pasta sauce with a gentleness that evokes her relationship with her children. She brings the final dishes to her children in the living space, before the viewer sees the three figures enjoy the meal together. This final scene suggests that nothing could be more enjoyable than cooking, sharing and eating a meal, containing meat, with one’s family in the comfort of the domestic space.

### Indulgence

Meat consumption can be rationalized by the belief that “the hedonistic pursuit of enjoyment and pleasure through ‘the good things in life’ is […] as an unassailable human right” (Arcari 2017) (p. 81). This attitude is discernible in Ramsay’s “perfect burger tutorial.”^20^ The idea of indulgence is evoked by several factors. The first is the setting: Ramsay is cooking outside, with blue skies and sunshine, next to a swimming pool, with the verdant, rolling hills of Los Angeles in the background. The second is his language: he explains that this is no ordinary burger, but his “F Word” burger, on sale at his restaurant in Las Vegas. By providing this recipe to his viewers, they have the opportunity to recreate this restaurant-quality “burger to die for” at home. Third, the burgers’ appearance: the beef patties – made of chuck, ground beef and brisket – are perfectly round, with neat, defined edges, well-seasoned and handled with care; they are then cooked on a high-end barbecue, basted with butter several times to enhance the taste through caramelization. The final factor is the consumer: having plated his burgers, Ramsay explains that his celebrity neighbors – Stevie Wonder, Kim Kardashian and Kanye West – enjoy coming round to his home to eat them, highlighting the burger’s status as an indulgent treat.

Whereas Ramsay’s burger evokes the idea of high-end restaurant indulgence, Halal Chef’s “Crispy chicken burger recipe”^21^ is more reflective of a chicken shop guilty pleasure. This can be seen in the opening clip, where zoomed-in shots of the final product are accompanied by “gangsta rap” music, a musical genre which, like the chicken shop, is largely associated with underprivileged, inner city areas (Tate 2013). The difference between the types of indulgence is enhanced by the location: in contrast to Ramsay’s Los Angeles pool-side barbecue station, Halal Chef greets the viewer from his British kitchen. Like Ramsay, however, he specifies what type of meat he is using, in this case chicken thighs. This suggests that knowledge of the cuts used in the dish increases its quality and, therefore, the pleasure it evokes in the consumer. The number of steps that go into preparing and cooking the chicken – removing excess fat, tenderizing, seasoning with spices, coating with flour, soaking in cold water, coating in flour (again), and deep frying – are also suggestive of a dish whose taste increases in correlation to the care and effort required to make it. When the dish is complete, the shot zooms in on Halal Chef taking a bite. The decadence is palpable in the ASMR (autonomous sensory meridian response (Poerio 2021) crunch of the chicken and Halal Chef’s satisfaction, perceptible both visibly and audibly. As a final indication of the superior taste of the burger, the video features a short clip of Ramsay saying his catchphrase: “f***ing delicious.” Halal Chef thus elevates chicken shop fare to an indulgent treat worthy of Ramsay and his celebrity neighbors. Both types of indulgence are pertinent with respect to South Asian British Muslims, with the rising demand for high-quality halal products on the one hand (Armanios and Ergene 2018) and the enduring association of fried chicken shops with halal cuisine on the other (Bagwell 2011).

## Discussion

### Mainstream meat

According to our data, between 2016 and 2020 uploads to YouTube of videos containing meat-based recipes overwhelmingly outnumbered vegetarian or vegan ones (73% versus 14%). This is consistent with other research findings examining television cookery programs in the UK: for example, only 10% of the savory recipes in the 2021 season of *MasterChef: The Professionals* were vegetarian or vegan (Booth 2021).

Our results are also consistent across the four chefs and halal recipes, though content was more meat-heavy for Ramsay and the halal search. Although Ramsay became a spokesperson for dairy-free milk brand Silk in 2021 (Webber 2021), he has traditionally been critical of plant-based eating: in 2016 he tweeted that he was allergic to “vegans” (Ramsay 2016), and in 2018 that he was a member of “PETA:” “People eating tasty animals” (Ramsay 2018). “Halal,” meanwhile, is a term typically associated with meat.

### Meat: healthy or unhealthy?

As to be expected in short recipe videos, where there is little space to explore food issues, we observed a clear absence of meat-related environment and animal welfare narratives, which correlates to the absenting of the moral and ethical issues around meat consumption. Although Mills (2017) notes that some food-related television programs “explore food sustainability and animal welfare, such as *Jamie’s Fowl Dinners*,” this stands apart from more specific cooking- and recipe-oriented content, which “invite[s] audiences to take pleasure in meat without considering the systems (industrial, cultural, moral) by which that meat comes to be” (p. 189–90).

Despite the lack of space for discussion, we observed the presence of some health-related narratives. This was usually in relation to chicken, and once to turkey and fish. Studies have observed white meat to be a healthier option than red and/or processed meat in relation to all-cause mortality (Etemadi et al. 2017). However, others have observed intake of all types of meat – including poultry – to increase the risk of NCDs like diabetes (Tonstad et al. 2013) and heart disease (Tong et al. 2019) in comparison to vegetarian/vegan diets. This scientific context is reflected in the nuances of the chicken-related health narratives, for example in Ramsay’s suggestion to his children that chicken is healthy, but not deep fried every night. It is also significant that no positive health narratives concerned red and processed meat, whose associations with increased risk of colorectal cancer (Bradbury, Murphy, and Key 2020) led the World Health Organization to classify processed meat as carcinogenic and red meat as “probably” carcinogenic (World Health Organization 2021).

### Normal, natural, necessary and nice

The idea of meat consumption as “normal” is clear in the high proportion of meat-based versus v/vg recipes, as well as in the absenting of meat’s animal origins. But it is also supported by Piazza et al. (2015)’s three other Ns that enable consumers to overcome cognitive dissonance. Meat is seen as a natural and necessary component of meals in its “defaultization.” This is evinced, first, by its addition to recipes that typically would not require it, such as vegetarian dishes, and second, by the lack of specificity, in some instances, regarding the type of meat used – the important thing is that *some* type of meat is present. Meat is also perceived as “nice,” being associated with positive emotional routines: it is enjoyed on social occasions, in the domestic space among family, and also as an indulgent treat – indeed, when animal body parts are mentioned, it usually relates to enhancing taste and, therefore, increasing pleasure. In the first scenario, meat could also be considered necessary both for cultural preservation and sociality, since “to deny the meat would be to also deny that sociality” (Arcari 2017) (p. 81). However, there is no indication that meat is necessary for health or survival (Piazza et al. 2015).

## Conclusions

In answer to our three research questions, we observe, first, that there is an overwhelmingly greater proportion of meat recipe videos to vegetarian or vegan ones. Secondly, although it was expected that environmental and animal welfare narratives would not feature in our sample of short recipe videos, given their usual absence in television programs which focus on cooking (Mills 2017), some health narratives around meat and plant-based consumption were present. Thirdly, by analyzing three main disengagement techniques present in these videos (the absenting of meat’s animal origins, the “defaultization” of meat as an ingredient, and evoking positive emotional routines), we see how these techniques serve to enhance the perception of meat consumption not only as “normal,” but also as “natural,” “necessary” and “nice” (Piazza et al. 2015).

To our knowledge this is the first study which examines the different ways that meat is normalized in audio-visual cookery content. A number of studies have explored the direct and indirect strategies that consumers adopt to justify eating meat (Piazza et al. 2015; Rothgerber 2013; Graça, Manuela Calheiros, and Oliveira 2014) and overcome the cognitive dissonance involved in the “meat paradox” (Bastian and Loughnan 2016; Loughnan, Haslam, and Bastian 2010). Whereas Arcari (2017) has examined how meat is normalized in international reports on the environment and sustainability, we used a multi-method approach to offer a detailed and nuanced examination of how meat is normalized in audio-visual content available on YouTube, with the selection of recent videos presented by a well-known presenters. Grounding the CDA theoretically in Piazza et al. (2015)’s concept of the 4 Ns facilitates discussion of how meat consumption is legitimized, and therefore normalized, in popular audio-visual cookery content.

Rather than focusing on recipe content generally available on YouTube, this paper focuses on content widely consumed by British Pakistani and Bangladeshi Muslims. As laid out in the Introduction, the two main reasons for this choice are that British Muslims eat more meat per capita than the UK average (Stannard and Clarke 2020), and that individuals from South Asian (and particularly Pakistani and Bangladeshi) backgrounds experience some of the poorest health outcomes in the UK (Raleigh and Holmes 2021), including diabetes (Sproston and Mindell 2006) and cardiovascular disease (Scarborough et al. 2010), associated with meat consumption (Tonstad et al. 2013; Tong et al. 2019). The presence of these health narratives even in short recipe videos consumed by British Muslims is therefore an important finding. For example, studies have shown that Bangladeshis in East London, despite being eager “to understand and explain the onset and experience” of diabetes (Greenhalgh, Helman, and Chowdhury 1998) (p. 980) and “to take personal responsibility for healthy lifestyle change” (Grace et al. 2008) (p. 6), often are not well supported by health professionals, who have been found to be “reluctant to discuss lifestyle change in clinical consultations […] because they perceived Bangladeshis as fatalistic […] and hence resistant to education on diabetes prevention” (Grace et al. 2008) (p. 4). This poses clear obstacles regarding the perceptions of health professionals and patient responsibility, and the rights of the patients to enquire about their illness.

The media plays an important role in the dissemination of health information to these groups. In October-December 2020, for example, social media was cited as a source of coronavirus-related information for 53% of Asian survey respondents, compared to 33% of White respondents (Ofcom 2018a, 2018b). This is a potentially powerful tool for two reasons. First, studies have shown common ethnicity to facilitate favorable perceptions toward celebrity trustworthiness (Lord, Putrevu, and Collins 2019). Second, British Bangladeshis have been found to turn less to professional or scientific explanations of diabetes, and more to the experiences of friends and family (Greenhalgh, Helman, and Chowdhury 1998); as such, if one member of an ethnic group watches a video featuring prominent voices from the community – which is probable, given the high use of YouTube among British Muslims (Stannard and Clarke 2020)—s/he is likely to pass the core messages on to others. Given the importance of television cookery programs and YouTube in influencing how individuals from British Pakistani and Bangladeshi backgrounds describe their eating habits, audio-visual recipe content that features trusted, ethnically congruous chefs and/or celebrities, who discuss the impact of diet on health – including the associations between meat and various non-communication diseases – could be considered as part of a multifarious strategy to improve health inequality in the UK.

## Supplementary Material

Supplemental Material

## Notes

1. This statistic was not included in the public report. The authors of this paper received permission from the report’s authors to quote this statistic.

2. The lower number for Lawson may be related to the nature of her channel, which sporadically uploads older recipe videos which previously featured on her television programmes. Though such recipe videos also appear on Oliver and Ramsay’s channels, both chefs regularly upload a large amount of new recipe content created specifically for YouTube.

3. https://tools.digitalmethods.net/netvizz/youtube/mod_videos_list.php.

4. https://www.youtube.com/watch?v=JWdTvyy6-mU.

5. https://www.youtube.com/watch?v=LG12Cg4Ag1g.

6. https://www.youtube.com/watch?v=5rJvTroqvKw.

7. https://www.youtube.com/watch?v=-K2WNtwbP2Y.

8. https://www.youtube.com/watch?v=FTociictyyE.

9. https://www.youtube.com/watch?v=jsWK0skZ0S8.

10. https://www.youtube.com/watch?v=MuajFTgkoHw.

11. https://www.youtube.com/watch?v=CkwflddQShk.

12. Ramsay is a notable exception. In his “Buttermilk Fried Chicken with Sweet Pickled Celery” recipe, he explains to his son, Jack, where the meat they are cooking comes from on the chicken by patting Jack on the corresponding limb (e.g., arm, leg). In another video featuring a clip from his butcher discussing poultry, Ramsay explains that “knowledge is crucial; the more you know about where your ingredients come from and how they’re produced, the better.” Not all of his recipes feature this sort of discussion, however, and for the most part, like the other chefs, he cooks meat without reference to its origins.

13. https://www.youtube.com/watch?v=qsVZKgt4Q10.

14. https://www.youtube.com/watch?v=mdYBS_qO9a4.

15. https://www.youtube.com/watch?v=DonJQk9_OBg.

16. https://www.youtube.com/watch?v=Mc-uMcoypZc.

17. https://www.youtube.com/watch?v=03h-RRm5RA8.

18. https://www.youtube.com/watch?v=5I0Eit1wD34.

19. https://www.youtube.com/watch?v=ze2KzMm7xE0.

20. https://www.youtube.com/watch?v=iM_KMYulI_s.

21. https://www.youtube.com/watch?v=wBpjdR0qXIE.
