# Supplementary material for: What’s cooking? The normalization of meat in YouTube recipe videos consumed by South Asian British Muslims
Source: Food Cult Soc. 2023 Apr 11;27(2):363–81. doi: 10.1080/15528014.2023.2196195 (PMC10878681; doi:10.1080/15528014.2023.2196195)
Supplement: Supplemental Material [file RFFC_A_2196195_SM2412.docx]

# Supplementary material

## Health narratives

Recipes that appear in more than one video are only tabulated once, but with reference to the second video in which they appear.

| **Channel** | **Title** | **URL** | **Date** | **Narratives** |
| --- | --- | --- | --- | --- |
| **Jamie Oliver** | | | | |
| Jamie Oliver | Healthy Black Bean Soup \| Jamie Oliver | https://www.youtube.com/watch?v=UrJOAURIM7w | 03/01/2016 | Title: healthy  Black bean soup: “really healthy, it’s got three of your five a day, and it’s packed full of the good stuff” |
| Jamie Oliver | Veggie Spaghetti Bolognese \| Super Food Family Classics \| Jamie Oliver | https://www.youtube.com/watch?v=CkwflddQShk | 07/08/2016 | Vegetarian Bolognese: “it’s really nutritious, it’s perfectly balanced, it’s three of your 5 fruit and veg a day, which is fantastic. And it’s bigging up wonderful ways to get your protein without eating meat” [also in *Pasta 7 Ways \| Jamie Oliver \| Megamix*] |
| Jamie Oliver | Pasta 7 Ways \| Jamie Oliver \| Megamix | Pasta 7 Ways \| Jamie Oliver \| Megamix | 14/06/2020 | Aubergine and ricotta pasta dish: “really healthy”; “it’s got all your food groups: dairy, protein, carbohydrates, veg” |
| **Gordon Ramsay** | | | | |
| Gordon Ramsay | Buttermilk Fried Chicken with Sweet Pickled Celery \| Gordon Ramsay | https://www.youtube.com/watch?v=JWdTvyy6-mU | 11/02/2017 | Fried chicken: explains to his children that “chicken is healthy, but not fried, every night, ok?” |
| The F Word | Gordon Ramsay's Chicken Parmesan Recipe: Extended Version \| Season 1 Ep. 3 \| THE F WORD | https://www.youtube.com/watch?v=IAbFlWQnlD4 | 15/06/2017 | Tenderstem broccoli: “healthy” |
| Gordon Ramsay | Gordon Ramsay Demonstrates Basic Cooking Skills \| Ultimate Cookery Course | https://www.youtube.com/watch?v=FTociictyyE | 07/11/2019 | Turkey: “a great lean meat […] anybody who’s worried about eating fatty meats, it’s a really healthy, flavoursome meal” [described by Ramsay’s butcher] |
| Gordon Ramsay | Quick & Simple Breakfast Recipes With Gordon Ramsay | https://www.youtube.com/watch?v=MuajFTgkoHw | 14/05/2020 | Bircher muesli dish: “[it] doesn’t just taste incredible, it makes you feel fantastic. It’s my ultimate healthy breakfast. There really is no better way to start the day.”  Yoghurt: “I think live and raw milk yoghurt are best. They’re packed with calcium, enzymes and probiotics, which help your body absorb nutrients”; “so much better for them [his kids]” than milk.  Oats: “low in the glycaemic index, which means they slowly release energy to keep you going right through to lunch”  Almonds: “incredibly healthy”  Berries: “don’t just taste great; they’re packed full of antioxidants and vitamins C and K”  Sprinkle mixture (black sesame seeds, chilli flakes, salt, pepper, lemon zest) for avocado toast: “healthy” |
| **Nigella Lawson** | | | | |
| Tonic | Nigella's Ultimate Comfort Food Recipes \| Nigella Bites \| Tonic | https://www.youtube.com/watch?v=LG12Cg4Ag1g | 25/09/2020 | Chicken soup: “There were some scientific tests of chicken soup recently which showed that it actually did possess anti-inflammatory and antibacterial properties […] you just have to taste this, even just look at it, to know that it’s going to do you good.” |
| **Nadiya Hussain** | | | | |
| This Morning | Nadiya Hussain's Healthy Chicken Shawarma \| This Morning | https://www.youtube.com/watch?v=5rJvTroqvKw | 15/07/2019 | Title: “healthy” |
| BBC | How to make a Salmon Poke Bowl in 15 minutes! \| Nadiya's Time to Eat - BBC | https://www.youtube.com/watch?v=jsWK0skZ0S8 | 10/08/2019 | Salmon dish: “nutritious” |
| **Halal recipes** | | | | |
| Immy Maryam | Weight Loss MEAL PREP \| How I LOST 20 Pounds FAST! ~ Immy | https://www.youtube.com/watch?v=-K2WNtwbP2Y | 19/05/2017 | Chicken and vegetable dish: “meal prep to lose weight and get shredded”; asks her viewers for “other healthy recipes”  Sweet potato: “our form of healthy carbohydrates for this meal” |
